# Plastome Phylogenomic and Biogeographical Study on *Thuja* (Cupressaceae)

**DOI:** 10.1155/2020/8426287

**Published:** 2020-06-26

**Authors:** Kole F. Adelalu, Xu Zhang, Xiaojian Qu, Jacob B. Landis, Jun Shen, Yanxia Sun, Aiping Meng, Hang Sun, Hengchang Wang

**Affiliations:** ^1^CAS, Key Laboratory of Plant Germplasm Enhancement and Specialty Agriculture, Wuhan Botanical Garden, Chinese Academy of Sciences, Wuhan 430074, China; ^2^Center of Conservation Biology, Core Botanical Gardens, Chinese Academy of Sciences, Wuhan, 430074 Hubei, China; ^3^University of Chinese Academy of Sciences, Beijing 100049, China; ^4^Key Lab of Plant Stress Research, College of Life Sciences, Shandong Normal University, Ji'nan, Shandong, China; ^5^Department of Botany and Plant Sciences, University of California Riverside, Riverside, CA, USA; ^6^School of Integrative Plant Science, Section of Plant Biology and the L.H. Bailey Hortorium, Cornell University, Ithaca, NY, USA; ^7^Key Laboratory for Plant Diversity and Biogeography of East Asia, Kunming Institute of Botany, Chinese Academy of Sciences, Kunming, Yunnan 650201, China

## Abstract

Investigating the biogeographical disjunction of East Asian and North American flora is key to understanding the formation and dynamics of biodiversity in the Northern Hemisphere. The small Cupressaceae genus *Thuja*, comprising five species, exhibits a typical disjunct distribution in East Asia and North America. Owing to obscure relationships, the biogeographical history of the genus remains controversial. Here, complete plastomes were employed to investigate the plastome evolution, phylogenetic relationships, and biogeographic history of *Thuja*. All plastomes of *Thuja* share the same gene content arranged in the same order. The loss of an IR was evident in all *Thuja* plastomes, and the B-arrangement as previously recognized was detected. Phylogenomic analyses resolved two sister pairs, *T*. *standishii*-*T*. *koraiensis* and *T*. *occidentalis*-*T*. *sutchuenensis*, with *T*. *plicata* sister to *T*. *occidentalis*-*T*. *sutchuenensis*. Molecular dating and biogeographic results suggest the diversification of *Thuja* occurred in the Middle Miocene, and the ancestral area of extant species was located in northern East Asia. Incorporating the fossil record, we inferred that *Thuja* likely originated from the high-latitude areas of North America in the Paleocene with a second diversification center in northern East Asia. The current geographical distribution of *Thuja* was likely shaped by dispersal events attributed to the Bering Land Bridge in the Miocene and subsequent vicariance events accompanying climate cooling. The potential effect of extinction may have profound influence on the biogeographical history of *Thuja*.

## 1. Introduction

Understanding the differences and connections of biogeographic distribution between the flora of the Northern Hemisphere, especially the flora of East Asia and North America, has been of great interest to systematists and biogeographers since the last century [1–8]. Multiple studies suggest that the geological activities and climatic oscillation in the Cenozoic, especially in the Late Neogene and Quaternary, have been responsible for plant biogeographic patterns of intercontinental disjunction [9–12]. In addition, two intercontinental land bridges, the Bering and North Atlantic Land Bridges, have played vital roles in the formation and dynamic floristic disjunctions due to their connectivity at different time points [4, 5, 8, 10]. Previous studies have shown that in angiosperm lineages, the prevalent pattern is origination in East Asia followed by migration to North America [13–16], while the opposite case, species originating in North America and then migrating to East Asia, has been reported in some gymnosperms [17–20]. Given that gymnosperms originated much earlier than angiosperms, they have been proposed as an ideal system for understanding deep biogeographical patterns, in particular those of an intercontinental nature.

*Thuja* is a small genus of Cupressaceae comprising five species: *T*. *plicata* Donn ex D. Don, *T*. *occidentalis* L., *T*. *koraiensis* Nakai, *T*. *standishii* (Gordon) Carrière, and *T*. *sutchuenensis* Franch [21, 22]. Species of *Thuja* are discontinuously distributed across East Asia and North America. Species *T*. *plicata* and *T*. *occidentalis* are distributed in western and eastern North America, respectively, whereas *T*. *koraiensis*, *T*. *standishii*, and *T*. *sutchuenensis* are endemic to East Asia with quite restricted distributions [23, 24].

The biogeographic history of *Thuja* remains controversial mainly due to the ambiguous relationships among species, despite several phylogenetic studies [23–25]. Early phylogenies of *Thuja*, including both the extant and fossil species, inferred that *T*. *sutchuenensis* may have arisen from an ancestor similar to the fossil species *T*. *polaris* and represents the earliest diverging clade of *Thuja* [25]. Molecular evidence from nrDNA ITS sequences of *Thuja* showed that *T*. *standishii* and *T*. *sutchuenensis* are sister and together they form a clade with *T*. *occidentalis*, while *T*. *koraiensis* and *T*. *plicata* form another clade. Based on the ITS tree, an eastern Asia origin and two dispersals to North America were inferred for *Thuja* [23]. Later, a phylogenetic study using five cpDNA regions (*rpl16*, *AtpI*-*rpoC1*, *trnS*-*trnfM*, *trnS*-*trnG*, and *trnT*-*trnF*), nrDNA ITS, and two low-copy nuclear genes (*LEAFY*, *4CL*) suggested high topological discordance between the chloroplast and nuclear gene trees, with discordance even occurring between the different nuclear gene trees [24]. In that study, the sister relationship of *T*. *sutchuenensis*-*T*. *standishii* was supported by both the chloroplast and nuclear genes, whereas the sister relationship of *T*. *koraiensis*-*T*. *plicata* was supported with *4CL*, nrDNA ITS, and combined nuclear gene trees. Biogeographic analysis based on the *4CL* tree indicated that the most recent common ancestor (MRCA) of *Thuja* likely had a wide distribution in East Asia and North America [24]. Additionally, comparisons of extant species and species known only in the fossil record showed that *Thuja* likely first appeared at high latitudes of North America before the Paleocene and spread to eastern Asia in the Miocene [26]. Owing to the relatively incompatible phylogenetic hypotheses proposed previously, the evolutionary history of *Thuja* still needs further investigation.

Phylogenomic approaches, which generate large amounts of DNA sequence data throughout the genome, have become increasingly essential for reconstructing entangled phylogenetic relationships among gymnosperms [27–29]. However, due to the extremely large genome sizes in gymnosperms [19, 30, 31], phylogenomic studies based on the nuclear genome still present significant challenges, most notably sequencing expense and annotation. In contrast, plastomes, which exhibit a high copy number per cell and a much smaller size, have been successfully implemented in phylogenetic inference of gymnosperms such as in the Pinaceae [32, 33] and Cupressaceae [34, 35]. Furthermore, the structural variations in plastomes are phylogenetically informative characters of themselves; for instance, the conifers (which *Thuja* belongs) are characterized by lacking canonical inverted repeats (IRs) and containing lineage-specific repeated tRNA genes [36–42]. Given the fundamental role of plastomes in understanding gymnosperm evolution, studies within each family/genus of conifers are insufficient to date.

In the present study, we newly sequenced three plastomes of *Thuja*. Including two previously reported plastomes [40, 43], we aim to (i) understand the plastome evolution of *Thuja*, (ii) reconcile the phylogenetic relationships within this small genus, and (iii) investigate the biogeographical history of *Thuja*. Additionally, rich molecular markers will be obtained as genetic resources for future research.

## 2. Results

### 2.1. Plastome Assembly, Structure, and Gene Content

**Figure 1:**
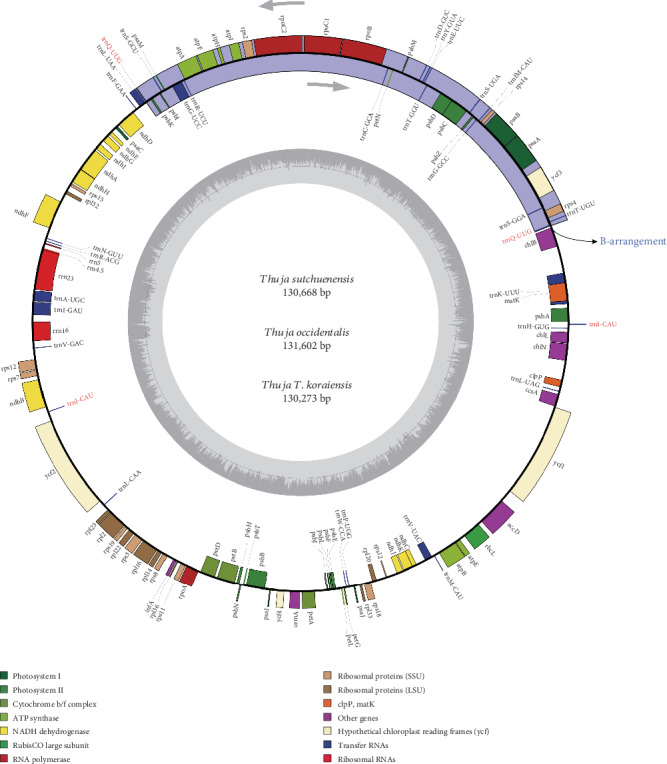
Graphic map of newly sequenced plastomes of *Thuja sutchuenensis*, *T*. *occidentalis*, and *T*. *koraiensis*. Genes transcribed clockwise are depicted on the inside of the circle, and genes transcribed counterclockwise are depicted on the outside. GC content is represented on the inner circle by dark gray bars. The location of the IR-mediated rearrangement is highlighted on the outer circle by blue bars.

**Figure 2:**
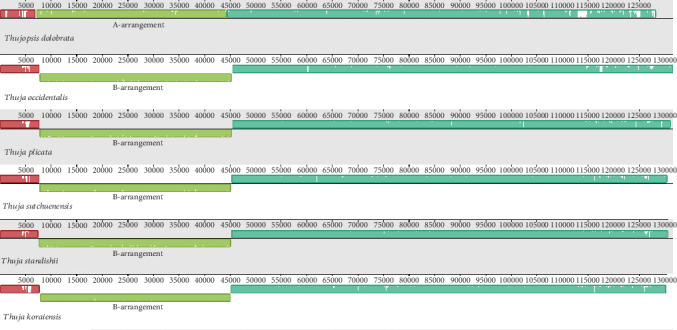
Mauve alignments with plastomes of *Thuja* and *Thujopsis*. The colored blocks represent collinear sequence blocks shared by all plastomes. The height of the colored bars within each block reflects the level of sequence similarity among plastomes. Two previously recognized rearrangements, A-rearrangement and B-rearrangement, are labeled.

After *de novo* and reference-guided assembly, we obtained three plastome sequences without gaps. The plastome features of all *Thuja* species are presented in Table 1. The size of *Thuja* plastomes ranges from 130,505 bp in *T*. *standishii* to 131,118 bp in *T*. *occidentalis*. A total of 116 genes are identified including 82 protein-coding genes, 4 ribosomal RNAs, and 30 transfer RNAs (Figure 1). Among the 116 unique genes, there are 15 genes that contain one intron (seven tRNA genes and eight protein-coding genes) and three protein-coding genes containing two introns (*rps12* and *ycf3*). Two tRNA genes, *trnI*-*CAU* and *trnQ*-*UUG*, have two copies (Table 2). Similar to other conifers, all species of *Thuja* are found to lack the inverted repeat (IR) region, thereby differing from the conventional quadripartite structure typical of angiosperm plastomes (Figure 1). In addition, a 36 kb inversion is detected in all *Thuja* plastomes, resulting in an isomeric plastome with the same B-arrangement as previously recognized [39]. This inversion segment is flanked by the duplicates of the *trnQ*-*UUG* gene (Figures 1 and 2).

### 2.2. Repetitive Sequences

**Figure 3:**
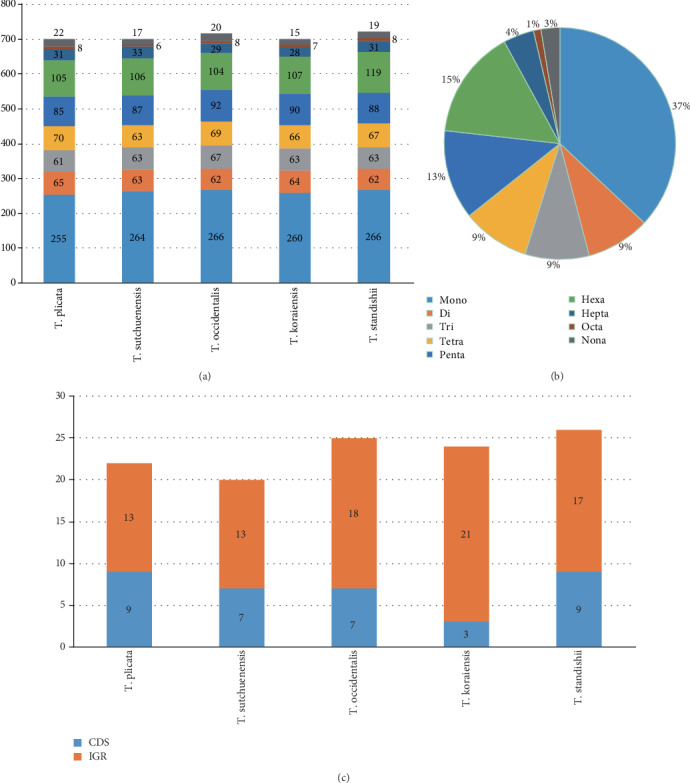
Information of simple sequence repeats (SSRs) and tandem repeats in *Thuja* plastomes. (a) Number of SSRs detected in the five *Thuja* plastomes. (b) Frequencies of identified SSR types in the five plastomes. (c) The number and distribution of tandem repeats in the five plastomes.

We characterized SSRs without setting a minimum satellite length constraint, thus obtaining abundant molecular markers. The numbers and types of SSRs are quite similar in all plastomes, with the total number ranging from 700 in *T*. *koraiensis* to 723 in *T*. *standishii* (Figure 3(a)). The proportion for each type of SSR is shown (Figure 3(b)). A total of 117 tandem repeats are detected in all five plastomes, with the number ranging from 20 in *T*. *sutchuenensis* to 26 in *T*. *standishii*. Among the 117 tandem repeats, 82 of them are located in intergenic regions (IGR) and 35 of them are in coding regions (CDS) (Figure 3(c)). The detailed information of SSRs and tandem repeats are provided in Supplementary Materials (Tables S2-S11).

### 2.3. Phylogenetic Analyses

**Figure 4:**
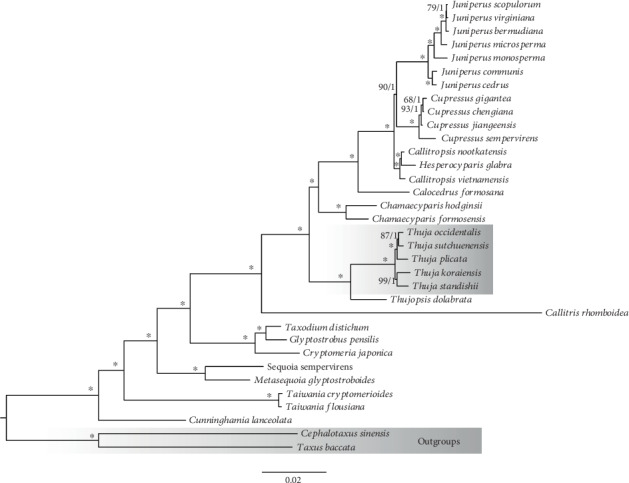
Phylogenomic results of *Thuja* from the ML (maximum likelihood) and BI (Bayesian inference) analyses using 82 protein-coding regions. Maximum likelihood bootstrap values (BS) and posterior probabilities (PP) are shown at nodes. Branches with ∗ have 100% BS and PP of 1.00.

After trimming poorly aligned fragments, the final alignment contained 82 protein-coding genes for 34 taxa consisting of 67,044 bp. The maximum likelihood (ML) and Bayesian inference (BI) analyses yielded identical tree topologies (Figure 4; BS value and PP are depicted in one tree). All nodes included in our phylogeny obtained robust supports. The sister relationship between *Thuja* and *Thujopsis* is supported with 100% bootstrap support and 1.0 posterior probability. Two clades are resolved within *Thuja* in the present phylogenetic analyses. *Thuja plicata* is sister to the clade formed by *T*. *sutchuenensis* and *T*. *occidentalis*, and they together are sister to the *T*. *standishii* and *T*. *koraiensis* alliance.

### 2.4. Divergence Time Estimation

**Figure 5:**
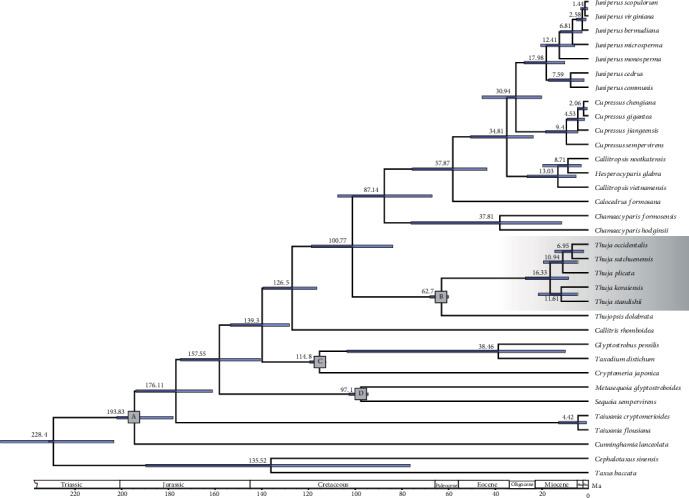
Divergence times of Cupressaceae estimated by BEAST with a relaxed molecular clock based on the combined protein-coding region sequences. A, B, C, and D indicate fossil calibration points. Median ages of nodes are shown with bars indicating the 95% highest posterior density intervals for each node.

The BEAST analysis based on 82 protein-coding regions yielded effective sample sizes that were well above 200 for all parameters, indicating adequate sampling of the posterior distribution. The MRCA of *Thuja* and *Thujopsis* was dated to 62.7 Ma (95% highest posterior density (HPD), 59.69-67.69 Ma). The divergence time among species of *Thuja* was estimated to be 16.33 Ma (95% HPD, 8.46-26.85 Ma). The time of MRCAs of the two recognized sister pairs, *T*. *standishii*-*T*. *koraiensis* and *T*. *occidentalis*-*T*. *sutchuenensis*, were approximately 11.61 Ma (95% HPD, 4.55-21.23 Ma) and 6.95 Ma (95% HPD, 2.05-14.29 Ma), respectively. The split time between *T*. *plicata* and *T*. *sutchuenensis*-*T*. *occidentalis* was estimated to be 10.94 Ma (95% HPD, 4.47-16.19 Ma; Figure 5).

### 2.5. Biogeographical Reconstruction of *Thuja*

**Figure 6:**
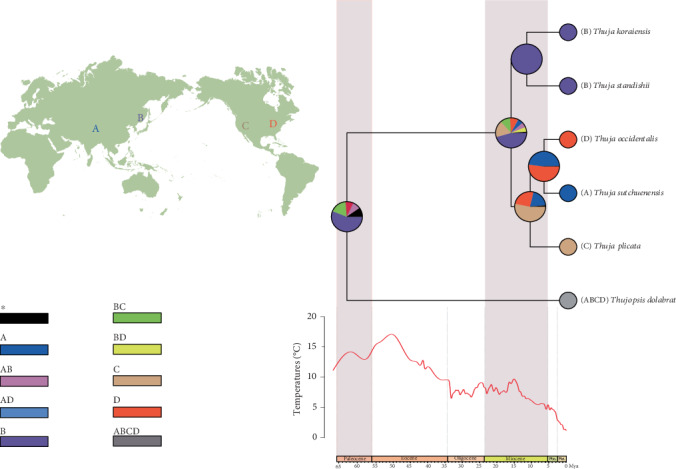
Ancestral reconstruction of *Thuja* by RASP and the corresponding global climate change over the past 65 million years (Zachos et al. 2001). (A) South West China (SW). (B) North East Asia (NE). (C) Western North America (WN). (D) Eastern North America (EN).

Model tests in BioGeoBEARS suggested that DEC+J is a better-performing model than the DEC model (AICc values: DEC+J = 30.2, DEC = 33.85, *p* = 0.0002). Although the application of the DEC+J model has been questioned previously [44], the selection of DEC+J may constitute the evidence in favor of founder-event speciation as a biogeographic process. The DEC+J analysis showed that the distribution area of *Thuja* is mostly restricted to northern East Asia (approx. 0.46) or less likely western North America (approx. 0.18) and northern East Asia/western North America (approx. 0.10). This may indicate that the MRCA of extant *Thuja* had a wide distribution in East Asia and North America. In addition, six dispersal and three vicariance events were identified within *Thuja* (Figure 6).

## 3. Discussion

### 3.1. Characterizations of *Thuja* Plastomes

Prior to this study, only two *Thuja* species had sequenced plastomes available [40, 42]. In the present study, we incorporated three newly sequenced plastomes with the two previously reported ones, which provided the opportunity to illustrate plastome evolution, as well as identify valuable molecular markers. All plastomes of *Thuja* possess 116 unique genes arranged in the same order, including 82 protein-coding genes, 30 tRNA genes, and 4 rRNA genes, with *trnI*-*CAU* and *trnQ*-*UUG* having two copies. Previous studies have commonly reported that the plastomes of conifers are usually characterized by the loss of an IR [36–42], which is different from the typical quadripartite structure shared by most angiosperm plastomes [45]. As expected, the loss of an IR was evident in all *Thuja* plastomes.

Previously, repeated tRNA genes in direct or inverted copies have been discovered in conifer plastomes [37–40, 44]. For example, the duplicated gene *trnI*-*CAU* was discovered in Pinaceae [33, 37], *trnQ*-*UUG* gene had two copies in cupressophytes [38], duplicated *trnQ*-*UUG* and *trnI*-*CAU* genes were reported in *Taxus* [46] and *Cryptomeria* [47], and three duplicated tRNA genes, *trnI*-*CAU*, *trnQ*-*UUG*, and *trnN*-*GUU*, were found in *Torreya* [44]. Consistent with previous studies, we detected two repeated tRNA genes, *trnI*-*CAU* and *trnQ*-*UUG*, existing in all *Thuja* plastomes. As proposed previously [37, 41, 44], these types of repeated tRNA genes in direct or inverted copies are likely the result of an incomplete loss of IR regions.

The isomeric plastomes formed by the repeated *trnQ*-*UUG* gene (i.e., the *trnQ*-IR arrangements) have been discovered in the *Cephalotaxus* (Cephalotaxaceae; [38]) and Cupressoideae species (Cupressaceae; [39, 40]). Guo et al. [39] recognized two types of isomeric plastome arrangements, the A-arrangement and B-arrangement, by the comparative and Southern blot analyses in *Juniperus*. Later, another two new types, C- and D-arrangements, were discovered by Qu et al. [40] in *Calocedrus*. Both studies suggested that the *trnQ*-IR may have promoted homologous recombination activity and is responsible for the presence of different isomeric forms. In our study, all *Thuja* plastomes contain the B-arrangement (Figures 1 and 2), which supports the hypothesis of Qu et al. [40] that the B-arrangement predominates in cupressophyte plastomes. Notably, we found that the relict species of *Thujopsis*, which is sister to *Thuja*, exhibits the A-arrangement, indicating the plastome rearrangement may have occurred multiple times during cupressophyte evolution. From all the evidence above, we can infer that the existence of isomeric plastomes might be a diagnostic feature in cupressophytes.

### 3.2. Phylogenetic Relationships and Evolutionary History of *Thuja*

The whole plastome sequence data used in our study yielded well-supported relationships among Cupressaceae, as well as within *Thuja*. Our phylogenomic analyses suggested that species from East Asia and North America are not monophyletic, respectively. Inconsistent with previous studies [23, 24], our results support two sister pairs, *T*. *standishii*-*T*. *koraiensis* and *T*. *occidentalis*-*T*. *sutchuenensis* (Figure 4). Our plastome tree is more similar to previous results based on plastid data [24]. We consider this discrepancy most likely due to the different types of markers used in previous phylogenetic inference compared to ours. The closely related affinity of *T*. *standishii*-*T*. *koraiensis* is reflected from geographic distribution, with *T*. *koraiensis* distributed in the Changbai Mountains of northeastern China and the Korean Peninsula, and *T*. *standishii* native to Japan [24]. These sister species were resolved as the early diverging clade of *Thuja* and originated in the Middle Miocene, which corresponds to the fossil records of *T*. *nipponica* found in both Akita County (NE Honshu) and Sikhote-Alin area of the Russian Far East during the Miocene [26]. The younger sister pair, *T*. *occidentalis*-*T*. *sutchuenensis*, displays an intercontinental disjunction. *T*. *sutchuenensis* was previously listed as an extinct species in the wild by the IUCN Species Survival Commission (SSC), while was rediscovered in 1999 in Chengkou, Chongqing, in the southwest of China [21, 24]. In the Late Pliocene, the fossil record of *T*. *sutchuenensis* was discovered in Shanxi Province, in northwest China. The northern Greenland cone-bearing material of *T*. *occidentalis* has been dated to the Late Pliocene to Pleistocene [26]. From paleobotanical and our phylogenomic evidence, we hypothesize that the ancestor of *T*. *occidentalis* and *T*. *sutchuenensis* had a widespread distribution in the high-latitude areas of the Northern Hemisphere but that climate cooling in the Late Neogene and Quaternary created a barrier separating populations on either side of the ocean. The barrier further facilitated the allopatric speciation of *Thuja*.

Prior to this study, two relatively incompatible standpoints have been proposed to explain the biogeographic process of *Thuja*. Li and Xiang [23] suggested an eastern Asia origin for *Thuja* based on ITS sequences. While the multiple gene evidence provided by Peng and Wang [24] indicated reticulate evolution occurring in *Thuja*, and they inferred that *Thuja* could have originated from the high-latitude areas of North America, although only the *4CL* gene was used. Comparative analyses using fossils suggest that *Thuja* likely first appeared at high latitudes of North America in or before the Paleocene and arrived in eastern Asia in the Miocene [24, 26]. According to the present study, the diversification of *Thuja* was dated to approximately the Middle Miocene and the ancestral area was located in northern East Asia, indicating a second diversification center of *Thuja* in northern East Asia. In the Middle Miocene, the Bering Land Bridge and the warm climate may have facilitated long distance dispersals (LDD) from East Asia to North America. Subsequently, climate cooling and drying after the Miocene, acting as a vicariance driver, forced a southward migration of *Thuja* and restricted species in their current distributions.

Previous studies of gymnosperm radiations have mostly inferred Oligocene-age crown groups [11, 18, 48–51], as is the case in cycads [51], Pinaceae [11], and Cupressaceae [18], indicating relatively recent diversification occurring in gymnosperms. The extinction following ancient origination may contribute to the young ages of most living gymnosperm clades. In conifer lineages, the evidence for widespread extinction and range shrinkage has been extensively reported. For example, Sequoioideae and Taxodioideae had a widespread distribution in the Northern Hemisphere in the Cretaceous and Paleogene, with Sequoioideae also found in Australia, but now are restricted to southern North America and East Asia [52]. Species of *Torreya* were widely distributed in the Northern Hemisphere during the Cretaceous but are now restricted to East Asia and North America [44, 53]. Other examples include the genera *Chamaecyparis* [54], *Austrocedrus* [55], and *Calocedrus* [56] having wider distributions in the past. Fossils of *Thuja* have been widely found in sediments of Paleocene to Pleistocene age in the Northern Hemisphere from 36.8°N to 86.3°N [26, 57], reflecting an early widespread distribution of *Thuja*. Climatic oscillations and glaciation in the Quaternary have reportedly eliminated many plant groups from Europe [4, 53]; this phenomenon is apparently applicable to the whole Northern Hemisphere flora that has been largely influenced by recent extinction. We speculate that the discrepancy between geographic distributions and phylogenetic relationships of *Thuja* can be attributed to extinction, a process blurs evolutionary history of species while is difficult to trace. Therefore, in the intercontinental disjunction context, we advocate that the potential effect of extinction should be reevaluated in the East Asia and North America flora, in particular, for the ancient rare gymnosperms.

## 4. Methods

### 4.1. Taxon Sampling, Chloroplast DNA Isolation, and High-Throughput Sequencing

Two previously reported plastomes (*Thuja plicata* and *T*. *standishii*) [40, 43] were downloaded from National Center for Biotechnology Information (NCBI) database. The fresh leaves of *T*. *sutchuenensis*, *T*. *occidentalis*, and *T*. *koraiensis* were collected from Wuhan Botanical Garden, Chinese Academy of Sciences; Atlantic Botanical Garden, USA; and Changbaishan Mts. National Reserve, Jilin, China, respectively. Total DNA was extracted using a modified CTAB protocol [58]. The purified DNA was used to construct Illumina Nextera XT libraries (Illumina, San Diego, CA, USA) following the manufacturer's instructions. DNA sequencing was performed on an Illumina MiSeq platform with paired-end 300 bp reads using V3 chemistry at Kunming Institute of Botany, China.

### 4.2. Plastome Assembly, Annotation, and Comparative Analyses

After sequencing, Illumina data were filtered using the NGS QC Toolkit (Patel and Jain 2012) by removing adapter sequences and low-quality reads with a quality value ≤ 20. The remaining high-quality reads were assembled into contigs with a minimum length of 1000 bp using SPAdes v.3.7.1 [59]. The complete plastome of *T*. *standishii* (GenBank: KX832627.1) was used as a reference for contig assembling [35]. Raw reads were then mapped against the resulting single contig to ensure no gaps remained using Geneious v.9.0.2 [60]. The assembled plastomes were annotated using Dual Organellar GenoMe Annotator (DOGMA) [61], with manual correction of gene start and stop codons. The tRNA genes were identified with tRNAscan-SE [62]. Graphical maps of the circular plastomes were visualized with OGDRAW [63].

To estimate locally collinear blocks (LCBs) among the examined plastomes, we performed whole-genome alignment using progressive Mauve implemented in Mauve v2.3.1 [64] with default parameters. Simple sequence repeats (SSRs) were identified using Phobos v.3.3.12 (http://www.rub.de/ecoevo/cm/cm_phobos.htm). The default settings in the perfect search function were used by setting a repeat unit size ranging from one to ten without setting a minimum satellite length constraint. Tandem repeats were identified with Tandem Repeats Finder (TRF) [65] with default parameter settings. The tandem repeat lengths were 20 bp or more with the minimum alignment score and maximum period size set as 50 and 500 (respectively), and the identity of repeats was set to 90%.

### 4.3. Phylogenetic Analyses

Coding sequence (CDS) of all 82 protein-coding genes was extracted from the five plastomes of *Thuja*, 27 other Cupressaceae species, and two species of Taxaceae (*Taxus baccata* and *Cephalotaxus sinensis*) [52]. Accession number and voucher information for each species are provided (Supplementary Materials: Table S1). The 82 genes from the 34 taxa were concatenated in PhyloSuite v.1.1.14 [66] and aligned using MAFFT v.7.22 [67] under “--auto” strategy and codon alignment mode. Ambiguously aligned fragments of the alignment were removed using Gblocks [68] with the following parameter settings: minimum number of sequences for a conserved/flank position (17/17), maximum number of contiguous nonconserved positions (8), minimum length of a block (10), and allowed gap positions (all).

Both the maximum likelihood (ML) and Bayesian inference (BI) analyses were conducted. ML analysis was performed in RAxML v.8.2.10 [69] under the general time-reversible substitution model with the gamma model of rate heterogeneity (GTR+G). Bootstrap support was estimated with 1000 bootstrap replicates. BI analysis was executed in MrBayes v.3.2.3 [70] with the same model as ML analysis (GTR+G). Two runs were conducted in parallel with four Markov chains (one cold and three heated), with each running for 2,000,000 generations from a random tree and sampled every 200 generations. The average standard deviation of split frequencies (<0.01) was used for checking convergence. After discarding the first 25% of the trees as burn-in, the remaining trees were used to construct majority-rule consensus trees and calculate the posterior probability (PP). The final trees were viewed using FigTree v.1.4.2 [71].

### 4.4. Divergence Time Estimation

Divergence times for *Thuja* were estimated using BEAST v.1.8.4 [72]. The molecular clock test was used to compare the ML value with and without the molecular clock constraints under the GTR model using MEGA X [73]. The null hypothesis of equal evolutionary rates throughout the tree was rejected (with clock, ln*L*: -39638711.585; without clock, ln*L*: -157069.417; *P* < 0.001). Thus, an uncorrelated lognormal relaxed-clock model with the birth-death process tree prior was implemented. The uncorrelated lognormal model allows uncertainty in the age of calibrations to be represented as prior distributions rather than as strict calibration/fixed points [71]. The Markov Chain Monte Carlo runs were set to 500 million generations with sampling every 5000 generations. Tracer v.1.7.1 [74] was used to assess the effective sample size (ESS > 200) of each parameter. After a burn-in of 25%, the maximum clade credibility (MCC) tree with median branch lengths and 95% highest posterior density (HPD) intervals on nodes was built using TreeAnnotator 2.1.3 [71].

According to the previous comprehensive biogeographic study of Cupressaceae [52], four calibration points were used: (A) the crown age of Cupressaceae, (B) the stem age of *Thuja*, (C) the split time among *Cryptomeria*, *Glyptostrobus*, and *Taxodium*, and (D) the MRCA of *Sequoia*-*Metasequoia*. These calibration points constrained the minimum age to 157.2 Ma, 58.5 Ma, 111 Ma, and 92.8 Ma, respectively. Following the study of Mao et al. [52], we modeled calibrated nodes with a lognormal distribution with a mean of 0.5, standard deviation of 1.5, and an offset (hardbound constraint) that equaled the minimum age of the calibrations.

### 4.5. Ancestral Area Reconstructions

To infer ancestral distribution ranges of living species of *Thuja*, we used the R package BioGeoBEARS (http://CRAN.Rproject.org/package=BioGeoBEARS), as implemented in program RASP v.4.0 [75]. A total of 10,000 random trees and one MCC tree generated by BEAST were used as input trees, which included only *Thuja* and its sister genus *Thujopsis*. We used the Dispersal-Extirpation-Cladogenesis (DEC) model [76], which allows dispersal, extinction, and cladogenesis as fundamental processes, accommodates differing dispersal probabilities among areas across different time periods, and can integrate branch lengths, divergence times, and geological information. We compared the DEC model with the “+J” suffix (i.e., DEC+J), which allows for founder speciation events. According to current distributions, species of *Thuja* were assigned to four possible geographic areas: (A) southwest region of China, (B) northern East Asia, (C) western North America, and (D) eastern North America. At most, two areas were allowed for any node in any tree, as each sampled taxon is restricted to only one area. Influenced by extinction, the relict distribution of *Thujopsis* may not be representative [24]; thus, we labeled it as an ambiguous geographic area (ABCD). An among-area dispersal probability matrix, which was inferred from the connectivity of the Bering Land Bridge [10], was coded to define different dispersal probabilities in four time periods, 0-5, 5-30, 30-45, and 45-65 (Table S12).

## 5. Conclusions

In the present study, we sequenced and analyzed complete plastomes of *Thuja*, providing new insight into plastome evolution, phylogenetic relationships, and evolutionary history. Phylogenomic analyses based on plastome sequences yielded robust relationships within *Thuja*. Incorporating paleobotanical evidence, we hypothesize a North American origination and a northern East Asia diversification of *Thuja*. The current geographical distribution of *Thuja* was likely shaped by dispersal events attributed to the Bering Land Bridge in the Miocene and subsequent vicariance events accompanying climate cooling. We further inferred that the potential effect of extinction has had profound influence on the biogeographical history of *Thuja*. Our study highlights the utility of plastome-scale datasets in resolving controversial phylogeny and inferring biogeographical history.

## Figures and Tables

**Table 1:** Comparison of plastomes within *Thuja*.

Species	Size (bp)	Total number of genes	Ribosomal RNA	Transfer RNAs	Protein-coding genes	GC content (%)	Protein-coding genes (bp)	Ribosomal RNAs (bp)	Transfer RNAs (bp)
*T*. *plicata*	131,118	116	4	30	82	34.30	75,663	4,796	2,341
*T*. *sutchuenensis*	130,668	116	4	30	82	35.81	75,392	4,479	2,286
*T*. *occidentalis*	131,602	116	4	30	82	34.26	76,210	4,479	2,413
*T*. *koraiensis*	130,273	116	4	30	82	34.30	74,271	4,478	2,475
*T*. *standishii*	130,505	116	4	30	82	34.24	75,333	4,874	2,413

**Table 2:** List of genes identified in the plastomes of *Thuja*.

Functional groups	Group of genes	Names of genes
Protein synthesis and DNA replication	Ribosomal RNAs	*rrn5*, *rrn4*.*5*, *rrn23*, *rrn16*
	Transfer RNAs	*trnH*-*GUG*, *trnI*-*CAU^+^*, *trnK–UUU*^∗^, *trnQ*-*UUG^+^*, *trnT*-*UGU*, *trnS*-*GGA*, *trnfM*-*CAU*, *trnG*-*GCC*, *trnS*-*UGA*, *trnT*-*GGU*, *trnE*-*UUC*, *trnY*-*GUA*, *trnD*-*GUC*, *trnC*-*GCA*, *trnR*-*UCU*, *trnG*-*UCC*^∗^, *trnS*-*GCU*, *trnL*-*UAA*^∗^, *trnF*-*GAA*, *trnN*-*GUU*, *trnR*-*ACG*, *trnA*-*UGC*^∗^, *trnI*-*GAU*^∗^, *trnV*-*GAC*, *trnL*-*CAA*, *trnW*-*CCA*^∗^, *trnP*-*UGG*, *trnV*-*UAC*^∗^, *trnM*-*CAU*, *trnL*-*UAG*
	Small subunit	*rps3*, *rps7*, *rps8*, *rps11*, *rps18*, *rps19*, *rps4*, *rps14*, *rps2*, *rps15*, *rps12*^∗^
	Ribosomal proteins large subunit	*rpl32*, *rpl23*, *rpl2*^∗^, *rpl22*, *rpl16*^∗^, *rpl14*, *rpl36*, *rpl33*, *rpl20*
	RNA polymerase	*rpoB*, *rpoC1*^∗^, *rpoC2*, *rpoA*

Photosynthesis	Photosystem I	*psaA*, *psaB*, *psaM*, *psaC*, *psaI*, *psaJ*
	Photosystem II	*psbA*, *psbD*, *psbZ*, *psbC*, *psbM*, *psbI*, *psbK*, *psbH*, *psbN*, *psbT*, *psbB*, *psbJ*, *psbL*, *psbF*, *psbE*
	Cytochrome b6/f	*petN*, *petD*^∗^, *petB*^∗^, *petA*, *petL*, *petG*
	ATP synthase	*atpI*, *atpH*, *atpF*^∗^, *atpA*, *atpB*, *atpE*
	NADH dehydrogenase	*ndhD*, *ndhE*, *ndhG*, *ndhI*, *ndhA*^∗^, *ndhH*, *ndhF*, *ndhB*^∗^, *ndhJ*, *ndhK*, *ndhC*
	Large subunit of RuBisCO	*rbcL*

Miscellaneous proteins	Subunit of acetyl-CoA-carboxylase c-type cytochrome synthesis gene	*accD*, *ccsA*, *cemA*, *clpP*^∗∗^, *infA*, *chlB*, *chlL*, *chlN*, *matK*

Genes of unknown function	Hypothetical conserved coding frame	*ycf3* ^∗∗^, *ycf2*, *ycf4*, *ycf1*

^∗^Gene containing a single intron. ^∗∗^Gene containing two introns. ^+^Gene having two copies.

## Data Availability

The newly sequenced plastomes have been submitted to GenBank; accession numbers are provided in Table S1 (Additional files).

## Supplementary Materials

Supplementary Materials There are additional tables in the supplementary materials file. Table S1: the taxa information including GenBank accession numbers used in the present study. Tables S2-S12: the distributions of tandem repeats and characteristics of simple sequence repeats (SSRs) identified in the plastomes of Thuja species. For each table, there is a detailed title included in the supplementary materials.

## References

[1] Li H.-L. (1952). Floristic relationships between eastern Asia and eastern North America. *Transactions of the American Philosophical Society*.

[2] Boufford D. E., Spongberg S. A. (1983). Eastern Asian-Eastern North American Phytogeographical Relationships-A History From the Time of Linnaeus to the Twentieth Century. *Annals of the Missouri Botanical Garden*.

[3] Zhengyi W. (1983). On the Significance of Pacific Intercontinental Discontinuity. *Annals of the Missouri Botanical Garden*.

[4] Tiffney B. H. (1985). Perspectives on the origin of the floristic similarity between Eastern Asia and Eastern North America. *Journal of the Arnold Arboretum*.

[5] Donoghue M. J., Bell C. D., Li J. H. (2001). Phylogenetic patterns in Northern Hemisphere plant geography. *International Journal of Plant Sciences*.

[6] Xiang Q. Y., Soltis D. E. (2001). Dispersal‐Vicariance Analyses of Intercontinental Disjuncts: Historical Biogeographical Implications for Angiosperms in the Northern Hemisphere. *International Journal of Plant Sciences*.

[7] Nie Z. L., Sun H., Li H., Wen J. (2006). Intercontinental biogeography of subfamily Orontioideae (*Symplocarpus*, *Lysichiton*, and *Orontium*) of Araceae in Eastern Asia and North America. *Molecular Phylogenetics and Evolution*.

[8] Wen J., Nie Z. L., Ickert-Bond S. M. (2016). Intercontinental disjunctions between eastern Asia and western North America in vascular plants highlight the biogeographic importance of the Bering Land Bridge from late Cretaceous to Neogene. *Journal of Systematics and Evolution*.

[9] Xiang Q. Y., Soltis D. E., Soltis P. S., Manchester S. R., Crawford D. J. (2000). Timing the eastern Asian-eastern North American floristic disjunction: molecular clock corroborates paleontological estimates. *Molecular Phylogenetics and Evolution*.

[10] Wen J. (2001). Evolution of Eastern Asian–Eastern North American biogeographic disjunctions: a few additional issues. *International Journal of Plant Sciences.*.

[11] Wei X. X., Yang Z. Y., Li Y., Wang X. Q. (2010). Molecular phylogeny and biogeography of *Pseudotsuga* (Pinaceae): insights into the floristic relationship between Taiwan and its adjacent areas. *Molecular Phylogenetics and Evolution*.

[12] Leslie A. B., Beaulieu J. M., Rai H. S., Crane P. R., Donoghue M. J., Mathews S. (2012). Hemisphere-scale differences in conifer evolutionary dynamics. *Proceedings of the National Academy of Sciences of the United States of America*.

[13] Oh S. H., Potter D. (2005). Molecular phylogenetic systematics and biogeography of tribe Neillieae (Rosaceae) using DNA sequences of cpDNA, rDNA, and *LEAFY*. *American Journal of Botany*.

[14] Ickert-Bond S. M., Wen J. (2006). Phylogeny and biogeography of Altingiaceae: evidence from combined analysis of five non-coding chloroplast regions. *Molecular Phylogenetics and Evolution*.

[15] Zhang M. L., Uhink C. H., Kadereit J. W. (2007). Phylogeny and biogeography of *Epimedium/Vancouveria* (Berberidaceae): western North American - East Asian disjunctions, the origin of European mountain plant taxa, and East Asian species diversity. *Systematic Botany.*.

[16] Wang H. D., Zheng J. H., Deng C. L., Liu Q. Y., Yang S. L. (2008). Fat embolism syndromes following liposuction. *Aesthetic plastic surgery.*.

[17] Ran J. H., Wei X. X., Wang X. Q. (2006). Molecular phylogeny and biogeography of *Picea* (Pinaceae): implications for phylogeographical studies using cytoplasmic haplotypes. *Molecular Phylogenetics and Evolution*.

[18] Mao K., Hao G., Liu J., Adams R. P., Milne R. I. (2010). Diversification and biogeography of *Juniperus* (Cupressaceae): variable diversification rates and multiple intercontinental dispersals. *The New Phytologist*.

[19] Wang X. Q., Ran J. H. (2014). Evolution and biogeography of gymnosperms. *Molecular Phylogenetics and Evolution*.

[20] Ran J. H., Shen T. T., Liu W. J., Wang P. P., Wang X. Q. (2015). Mitochondrial introgression and complex biogeographic history of the genus *Picea*. *Molecular Phylogenetics and Evolution*.

[21] Xiang Q., Fajon A., Li Z., Fu L., Liu Z. (2002). Thuja sutchuenensis: a rediscovered species of the Cupressaceae. *Botanical Journal of the Linnean Society*.

[22] Farjon A. (2005). *A Monograph of Cupressaceae and Sciadopitys*.

[23] Li J. H., Xiang Q. P. (2005). Phylogeny and biogeography of Thuja L. (Cupressaceae), an eastern Asian and North American disjunct genus. *Journal of Integrative Plant Biology.*.

[24] Peng D., Wang X. Q. (2008). Reticulate evolution in *Thuja* inferred from multiple gene sequences: implications for the study of biogeographical disjunction between eastern Asia and North America. *Molecular Phylogenetics and Evolution*.

[25] Mciver E. E., Basinger J. F. (1989). The morphology and relationships ofThuja polarissp.nov. (Cupressaceae) from the Early Tertiary, Ellesmere Island, Arctic Canada. *Canadian Journal of Botany*.

[26] Cui Y. M., Sun B., Wang H. F. (2015). Exploring the formation of a disjunctive pattern between Eastern Asia and North America based on fossil evidence from *Thuja* (Cupressaceae). *PLoS One*.

[27] Soltis P. S., Soltis D. E. (2013). A conifer genome spruces up plant phylogenomics. *Genome Biology*.

[28] Mao K., Ruhsam M., Ma Y. (2019). A transcriptome-based resolution for a key taxonomic controversy in Cupressaceae. *Annals of Botany*.

[29] Ran J.-H., Shen T.-T., Wang M.-M., Wang X.-Q. (2018). Phylogenomics resolves the deep phylogeny of seed plants and indicates partial convergent or homoplastic evolution between Gnetales and angiosperms. *Proceedings of the Royal Society B: Biological Sciences.*.

[30] Rigault P., Boyle B., Lepage P., Cooke J. E. K., Bousquet J., MacKay J. J. (2011). A white spruce gene catalog for conifer genome analyses. *Plant Physiology*.

[31] Nystedt B., Street N. R., Wetterbom A. (2013). The Norway spruce genome sequence and conifer genome evolution. *Nature*.

[32] Parks M., Cronn R., Liston A. (2009). Increasing phylogenetic resolution at low taxonomic levels using massively parallel sequencing of chloroplast genomes. *BMC Biology*.

[33] Lin C. P., Huang J. P., Wu C. S., Hsu C. Y., Chaw S. M. (2010). Comparative chloroplast genomics reveals the evolution of Pinaceae genera and subfamilies. *Genome Biology and Evolution*.

[34] Zhu A., Fan W., Adams R. P., Mower J. P. (2018). Phylogenomic evidence for ancient recombination between plastid genomes of the *Cupressus*-*Juniperus*-*Xanthocyparis* complex (Cupressaceae). *BMC Evolutionary Biology*.

[35] Qu X.-J., Jin J.-J., Chaw S.-M., Li D.-Z., Yi T.-S. (2017). Multiple measures could alleviate long-branch attraction in phylogenomic reconstruction of Cupressoideae (Cupressaceae). *Scientific Reports*.

[36] Raubeson L. A., Jansen R. K. (1992). A rare chloroplast-DNA structural mutation is shared by all conifers. *Biochemical Systematics and Ecology.*.

[37] Wu C. S., Wang Y. N., Hsu C. Y., Lin C. P., Chaw S. M. (2011). Loss of different inverted repeat copies from the chloroplast genomes of Pinaceae and cupressophytes and influence of heterotachy on the evaluation of gymnosperm phylogeny. *Genome Biology and Evolution*.

[38] Yi X., Gao L., Wang B., Su Y. J., Wang T. (2013). The Complete Chloroplast Genome Sequence of Cephalotaxus oliveri (Cephalotaxaceae): Evolutionary Comparison of Cephalotaxus Chloroplast DNAs and Insights into the Loss of Inverted Repeat Copies in Gymnosperms. *Genome Biology and Evolution*.

[39] Guo W., Grewe F., Cobo-Clark A. (2014). Predominant and substoichiometric isomers of the plastid genome coexist within *Juniperus* plants and have shifted multiple times during cupressophyte evolution. *Genome Biology and Evolution*.

[40] Qu X. J., Wu C. S., Chaw S. M., Yi T. S. (2017). Insights into the existence of isomeric Plastomes in Cupressoideae (Cupressaceae). *Genome Biology and Evolution*.

[41] Chaw S.-M., Wu C.-S., Sudianto E., Chaw S.-M., Jansen R. K. (2018). Evolution of Gymnosperm Plastid Genomes. *In: Plastid Genome Evolution*.

[42] Zhang X., Zhang H. J., Landis J. B. (2019). Plastome phylogenomic analysis ofTorreya(Taxaceae). *Journal of Systematics and Evolution*.

[43] Adelalu K. F., Qu X. J., Sun Y. X., Deng T., Sun H., Wang H. C. (2019). Characterization of the complete plastome of western red cedar, *Thuja plicata* (Cupressaceae). *Conservation Genetics Resources.*.

[44] Ree R. H., Sanmartín I. (2018). Conceptual and statistical problems with the DEC+J model of founder-event speciation and its comparison with DEC via model selection. *Journal of Biogeography*.

[45] Palmer J. D. (1985). Comparative organization of chloroplast genomes. *Annual Review of Genetics*.

[46] Zhang Y., Ma J., Yang B. (2014). The complete chloroplast genome sequence of *Taxus chinensis* var. *mairei* (Taxaceae): loss of an inverted repeat region and comparative analysis with related species. *Gene*.

[47] Hirao T., Watanabe A., Kurita M., Kondo T., Takata K. (2008). Complete nucleotide sequence of the Cryptomeria japonica D. Don. chloroplast genome and comparative chloroplast genomics: diversified genomic structure of coniferous species. *BMC Plant Biology*.

[48] Won H., Renner S. S. (2006). Dating dispersal and radiation in the gymnosperm *Gnetum* (Gnetales)--clock calibration when outgroup relationships are uncertain. *Systematic Biology*.

[49] Ickert-Bond S. M., Rydin C., Renner S. S. (2009). A fossil-calibrated relaxed clock forEphedraindicates an Oligocene age for the divergence of Asian and New World clades and Miocene dispersal into South America. *Journal of Systematics and Evolution*.

[50] Biffin E., Hill R. S., Lowe A. J. (2010). Did Kauri (*Agathis*: Araucariaceae) really survive the Oligocene drowning of New Zealand?. *Systematic Biology*.

[51] Nagalingum N. S., Marshall C. R., Quental T. B., Rai H. S., Little D. P., Mathews S. (2011). Recent synchronous radiation of a living fossil. *Science*.

[52] Mao K., Milne R. I., Zhang L. (2012). Distribution of living Cupressaceae reflects the breakup of Pangea. *Proceedings of the National Academy of Sciences of the United States of America*.

[53] Li J., Davis C. C., Donoghue M. J., Kelley S., Del Tredici P. (2001). Phylogenetic relationships of *Torreya* (Taxaceae) inferred from sequences of nuclear ribosomal DNA ITS region. *Harvard Papers in Botany*.

[54] Liu Y.-S., Mohr B. A. R., Basinger J. F. (2009). Historical biogeography of the genus Chamaecyparis (Cupressaceae, Coniferales) based on its fossil record. *Palaeobiodiversity and Palaeoenvironments.*.

[55] Paull R., Hill R. S. (2008). OligoceneAustrocedrusfrom Tasmania (Australia): comparisons *withAustrocedrus chilensis*. *International Journal of Plant Sciences.*.

[56] Shi G., Zhou Z., Xie Z. (2012). A new Oligocene Calocedrus from South China and its implications for transpacific floristic exchanges. *American Journal of Botany*.

[57] Sun B., Cui Y. M., Wang H. F. (2015). Recognizing the species of Thuja (Cupressaceae) based on their cone and foliage morphology. *Phytotaxa.*.

[58] Yang J. B., Li D. Z., Li H. T. (2014). Highly effective sequencing whole chloroplast genomes of angiosperms by nine novel universal primer pairs. *Molecular Ecology Resources*.

[59] Bankevich A., Nurk S., Antipov D. (2012). SPAdes: a new genome assembly algorithm and its applications to single-cell sequencing. *Journal of Computational Biology*.

[60] Kearse M., Moir R., Wilson A. (2012). Geneious basic: an integrated and extendable desktop software platform for the organization and analysis of sequence data. *Bioinformatics*.

[61] Wyman S. K., Jansen R. K., Boore J. L. (2004). Automatic annotation of organellar genomes with DOGMA. *Bioinformatics*.

[62] Lowe T. M., Eddy S. R. (1997). tRNAscan-SE: a program for improved detection of transfer RNA genes in genomic sequence. *Nucleic Acids Research*.

[63] Lohse M., Drechsel O., Kahlau S., Bock R. (2013). OrganellarGenomeDRAW—a suite of tools for generating physical maps of plastid and mitochondrial genomes and visualizing expression data sets. *Nucleic Acids Research*.

[64] Darling A. E., Mau B., Perna N. T. (2010). progressiveMauve: multiple genome alignment with gene gain, loss and rearrangement. *PLoS One*.

[65] Benson G. (1999). Tandem repeats finder: a program to analyze DNA sequences. *Nucleic Acids Research*.

[66] Zhang D., Gao F., Li W. X. (2018). Phylo Suite: an integrated and scalable desktop platform for streamlined molecular sequence data management and evolutionary phylogenetics studies. *bioRxiv*.

[67] Katoh K., Standley D. M. (2013). MAFFT multiple sequence alignment software version 7: improvements in performance and usability. *Molecular Biology and Evolution*.

[68] Talavera G., Castresana J. (2007). Improvement of phylogenies after removing divergent and ambiguously aligned blocks from protein sequence alignments. *Systematic Biology*.

[69] Stamatakis A. (2014). RAxML version 8: a tool for phylogenetic analysis and post-analysis of large phylogenies. *Bioinformatics*.

[70] Huelsenbeck J. P., Ronquist F. (2001). MRBAYES: Bayesian inference of phylogenetic trees. *Bioinformatics*.

[71] Rambaut A., Drummond A. *FigTree, ver. 1.4. 2*.

[72] Drummond A. J., Rambaut A. (2007). BEAST: Bayesian evolutionary analysis by sampling trees. *BMC Evolutionary Biology.*.

[73] Kumar S., Stecher G., Li M., Knyaz C., Tamura K. (2018). MEGA X: molecular evolutionary genetics analysis across computing platforms. *Molecular Biology and Evolution*.

[74] Rambaut A., Drummond A. J., Xie D., Baele G., Suchard M. A. (2018). Posterior summarization in Bayesian phylogenetics using tracer 1.7. *Systematic Biology*.

[75] Yu Y., Harris A. J., Blair C., He X. (2015). RASP (Reconstruct Ancestral State in Phylogenies): a tool for historical biogeography. *Molecular Phylogenetics and Evolution*.

[76] Ree R. H., Smith S. A. (2008). Maximum likelihood inference of geographic range evolution by dispersal, local extinction, and cladogenesis. *Systematic Biology*.

