# Supplementary material for: Plastome Phylogenomic and Biogeographical Study on Thuja (Cupressaceae)
Source: Biomed Res Int. 2020 Jun 26;2020:8426287. doi: 10.1155/2020/8426287 (PMC7335403; doi:10.1155/2020/8426287)
Supplement: Supplementary Materials — There are additional tables in the supplementary materials file. Table S1: the taxa information including GenBank accession numbers used in the present study. Tables S2-S12: the distributions of tandem repeats and characteristics of simple sequence repeats (SSRs) identified in the plastomes of Thuja species. For each table, there is a detailed title included in the supplementary materials. [file 8426287.f1.docx]

**Table S1** Taxa included in present study. Information including GenBank accession numbers are provided

| Species | Family | GenBank Accession number | Size (bp) | Total number of genes | GC content (%) |
| --- | --- | --- | --- | --- | --- |
| *Thuja plicata* | Cupressaceae | KY290451.1 | 131,118 | 117 | 34.30 |
| *Thuja sutchuenensis* | Cupressaceae | KY272867.1 | 130,668 | 116 | 35.81 |
| *Thuja occidentalis* | Cupressaceae | KY295906.1 | 131,602 | 116 | 34.26 |
| *Thuja koraiensis* | Cupressaceae | MG012464.1 | 130,273 | 116 | 34.30 |
| *Thuja standishii* | Cupressaceae | KX832627.1 | 130,505 | 116 | 34.24 |
| *Callitris rhomboidea* | Cupressaceae | NC_034940.1 | 121,117 | 110 | 34.71 |
| *Callitropsis nootkatensis* | Cupressaceae | KP099642.1 | 127,150 | 117 | 34.73 |
| *Callitropsis vietnamensis* | Cupressaceae | KP099645.1 | 127,541 | 115 | 34.73 |
| *Calocedrus formosana* | Cupressaceae | NC_023121.1 | 127,311 | 114 | 34.83 |
| *Calocedrus macrolepsis* | Cupressaceae | KX832621.1 | 127,157 | 118 | 34.86 |
| *Chamaecyparis hodginsii* | Cupressaceae | KX832623.1 | 128,789 | 118 | 34.89 |
| *Chamaecyparis formosensis* | Cupressaceae | NC_034943.1 | 127,211 | 117 | 34.99 |
| *Cryptomeria japonica* | Cupressaceae | NC_010548.1 | 131,810 | 117 | 35.38 |
| *Cunninghamia lanceolata* | Cupressaceae | NC_021437.1 | 135,334 | 112 | 35.00 |
| *Cupressus gigantea* | Cupressaceae | NC_028155.1 | 128244 | 115 | 34.71 |
| *Cupressus sempervirens* | Cupressaceae | KP099643.1 | 129,150 | 115 | 34.60 |
| *Cupressus chengiana* | Cupressaceae | NC_034788.1 | 128,151 | 117 | 34.73 |
| *Glyptostrobus pensilis* | Cupressaceae | NC_031354.1 | 132,239 | 117 | 35.30 |
| *Hesperocyparis glabra* | Cupressaceae | KP099644.1 | 126,993 | 116 | 34.69 |
| *Juniperus bermudiana* | Cupressaceae | NC_024021.1 | 127,659 | 117 | 34.84 |
| *Juniperus cedrus* | Cupressaceae | NC_028190.1 | 127,126 | 116 | 34.97 |
| *Juniperus cummunis* | Cupressaceae | NC_035068.1 | 128,334 | 113 | 34.86 |
| *Juniperus monosperma* | Cupressaceae | NC_024022.1 | 127,744 | 117 | 34.88 |
| *Juniperus scopulorum* | Cupressaceae | NC_024023.1 | 127,774 | 117 | 34.82 |
| *Juniperus virginiana* | Cupressaceae | NC_024024.1 | 127,770 | 117 | 34.83 |
| *Metasequoia glyptostroboides* | Cupressaceae | KR061358.1 | 131,887 | 116 | 35.25 |
| *Sequoia sempervirens* | Cupressaceae | NC_030372.1 | 133,929 | 112 | 35.37 |
| *Taiwania flousiana* | Cupressaceae | NC_021441.1 | 131,413 | 114 | 34.72 |
| *Taiwania cryptomenioides* | Cupressaceae | NC_016065.1 | 132,588 | 118 | 34.63 |
| *Platycladus orientallis* | Cupressaceae | KX832626.1 | 127,113 | 118 | 34.74 |
| *Taxodium distichum* | Cupressaceae | NC_034941.1 | 131,954 | 116 | 35.26 |
| *Thujopsis dolobrata* | Cupressaceae | KX832628.1 | 128,291 | 117 | 34.75 |
| *Taxus baccata* | Taxaceae | NC_035066 | 128,653 | 114 | 34.65 |
| *Cephalotaxus sinensis* | Taxaceae | MG385662 | 135,646 | 114 | 35.15 |

**Table S2** Distribution of tandem repeats in the *Thuja plicata*

|  | Indices | Size of repeat unit X Copy number | Repeat length | Location |
| --- | --- | --- | --- | --- |
| 1 | 7081-7118 | 20x2 | 40 | trnK-UUU(IGR/ psbD(CDS) |
| 2 | 9644-9675 | 16x2 | 32 | trnQ-UUG/trnT-UGU (IGR) |
| 3 | 14307-14343 | 18x2 | 36 | ycf3/ psaA (IGR) |
| 4 | 27463-27487 | 12x2 | 24 | petN/ trnC-GCA (IGR) |
| 5 | 45268-45307 | 19x2 | 38 | trnG-UCC/psaM (IGR) |
| 6 | 57061-57086 | 12x2 | 24 | rpl32/ndhF (IGR) |
| 7 | 75222-75558 | 67x5 | 335 | trnI-CAU/ ycf2(IGR) |
| 8 | 77314-77345 | 15x2 | 30 | ycf2 (CDS) |
| 9 | 79400-79442 | 21x2 | 42 | ycf2 (CDS) |
| 10 | 86427-86474 | 24x2 | 48 | rpl22 (CDS) |
| 11 | 90151-90213 | 24x2 | 48 | rps8/ infA (IGR) |
| 12 | 103537-103572 | 17x2 | 34 | psbE/petL (IGR) |
| 13 | 106097-106126 | 15x2 | 30 | rps18 (CDS) |
| 14 | 106215-106240 | 11x2 | 22 | rps18/ rpl20 (IGS) |
| 15 | 107689-107726 | 18x2 | 36 | rps12/ndhJ (IGR) |
| 16 | 113592-113631 | 20x2 | 40 | atpE/rbcL (IGR) |
| 17 | 116332-116363 | 15x2 | 30 | accD (CDS |
| 18 | 119938-119979 | 21x2 | 42 | ycf1 (CDS) |
| 19 | 121033-121089 | 27x2 | 54 | ycf1 (CDS) |
| 20 | 123265-123298 | 15x2 | 30 | ycf1 (CDS) |
| 21 | 128466-128528 | 24x2 | 48 | clpP/chlN (IGR) |
| 22 | 128487-128561 | 33x2 | 66 | clpP/ chlN(IGR) |

**Table: S3** Characteristics of simple sequence repeats identified in the plastomes of *T. plicata*

|  | Mono | Di | Tri | Tetra | Penta | Hexa | Hepta | Octa | Nona | Total |
| --- | --- | --- | --- | --- | --- | --- | --- | --- | --- | --- |
| Total counts | 255 | 65 | 61 | 70 | 85 | 105 | 31 | 8 | 22 | 702 |
| Total Repeat Length (repeat unit X number of repeat) (bp) | 2010 | 585 | 628 | 39 | 1010 | 1325 | 467 | 132 | 449 | 7345 |
| Density (Total repeat length/genome size) [bp/kb] | 15.33 | 4.46 | 4.79 | 5.64 | 7.70 | 10.11 | 3.56 | 1.01 | 3.42 | 56.02 |
| Proportion among other SSR (%) | 27.37 | 7.96 | 8.55 | 10.06 | 13.75 | 18.04 | 6.36 | 1.80 | 6.11 | 100 |
| Mean Length | 7.88 | 9.00 | 10.3 | 10.56 | 11.88 | 12.62 | 15.06 | 16.50 | 20.41 | 114.21 |

**Table S4** Distribution of tandem repeats in the *T. sutchuenensis*.

|  | Indices | Size of repeat unit X Copy number | Repeat length | Location |
| --- | --- | --- | --- | --- |
| 1 | 2187-2218 | 16x2 | 32 | trnQ-UUG/trnT-UGU (IGR) |
| 2 | 6830-6868 | 18x2 | 36 | ycf3/psaA (IGR) |
| 3 | 15961-16000 | 20x2 | 40 | psbD/ trnT-GGU(IGR) |
| 4 | 19995-20019 | 12x2 | 24 | petN/trnC-GCA (IGR) |
| 5 | 37817- 37856 | 19x2 | 28 | trnG-UCC/psaM (IGR) |
| 6 | 40675-40720 | 24x2 | 48 | trnL-UAA/trnF-GAA(IGR) |
| 7 | 49660-49685 | 12x2 | 24 | rpl32/ndhF (IGR) |
| 8 | 64570-64616 | 21x2 | 42 | rps7/ndhB (IGR) |
| 9 | 67833-67968 | 67x2 | 134 | trnI-CAU/ycf2 (IGR) |
| 10 | 69691-69722 | 15x2 | 30 | ycf2 (CDS) |
| 11 | 72181-72244 | 33x2 | 66 | ycf2 (CDS) |
| 12 | 78937-78973 | 18x2 | 36 | rpl22 (CDS) |
| 13 | 98326-98355 | 15x2 | 30 | rps18 (CDS) |
| 14 | 108569-108600 | 15x2 | 30 | accD (CDS) |
| 15 | 113283-113339 | 27x2 | 54 | ycf1 (CDS) |
| 16 | 115488-115521 | 15x2 | 30 | ycf1 (CDS) |
| 17 | 121406-121645 | 118x2 | 236 | clpP/chlN (IGR) |
| 18 | 121787-121821 | 18x2 | 36 | clpP/ chlN(IGR) |
| 19 | 124579-124605 | 13x2 | 26 | chlL/trnH-GUG (IGR) |
| 20 | 129697-129805 | 27x2 | 54 | trnK-UUU/chlB (IGR) |

**Table S5** Characteristics of simple sequence repeats identified in the plastomes of *T. sutchuenensis*

| Species | Mono | Di | Tri | Tetra | Penta | Hexa | Hepta | Octa | Nona | Total |
| --- | --- | --- | --- | --- | --- | --- | --- | --- | --- | --- |
| Total counts | 264 | 63 | 63 | 63 | 87 | 106 | 33 | 6 | 17 | 702 |
| Total Repeat Length (repeat unit X number of repeat) (bp) | 2091 | 568 | 650 | 662 | 1028 | 1339 | 490 | 100 | 357 | 7285 |
| Density (Total repeat length/genome size) [bp/kb] | 16.00 | 4.35 | 4.97 | 5.07 | 7.87 | 10.25 | 3.75 | 0.77 | 2.73 | 55.76 |
| Proportion among other SSR (%) | 28.70 | 7.80 | 8.92 | 9.09 | 14.11 | 18.38 | 6.73 | 1.37 | 4.90 | 100 |
| Mean Length | 7.92 | 9.02 | 10.32 | 10.51 | 11.82 | 12.63 | 14.85 | 16.67 | 21.00 | 114.74 |

**Table S6** Distribution of tandem repeats in the *Thuja occidentalis*

|  | Indices | Size of repeat unit X Copy number | Repeat length | Location |
| --- | --- | --- | --- | --- |
| 1 | 1920-1951 | 16x2 | 32 | trnQ-UUG/trnT-UGU (IGR) |
| 2 | 6589-6627 | 18x2 | 36 | ycf3/psaA (IGR) |
| 3 | 15721-15760 | 20x2 | 40 | psbD/trnT-GGU (IGR) |
| 4 | 15807-15844 | 19x2 | 38 | psbD/trnT-GGU (IGR) |
| 5 | 19786-19810 | 12x2 | 24 | petN/ trnC-GCA (IGR) |
| 6 | 30311-30376 | 12x5 | 60 | rpoC2 (CDS) |
| 7 | 37608-37647 | 19x2 | 38 | trnG-UCC/ psaM (IGR) |
| 8 | 49447-49472 | 12x2 | 24 | rpl32/ ndhF (IGR) |
| 9 | 54243-54510 | 65x4 | 260 | rrn5/ rrn4.5 (IGR) |
| 10 | 64516- 64562 | 21x2 | 42 | rps7/ ndhB (IGR) |
| 11 | 67779-68115 | 67x5 | 335 | trnI-CAU/ ycf2 (IGR) |
| 12 | 69838-69869 | 15x2 | 30 | ycf2 (CDS) |
| 13 | 72155-72197 | 21x2 | 42 | ycf2 (CDS) |
| 14 | 72433-72496 | 33x2 | 66 | ycf2 (CDS) |
| 15 | 82630-82901 | 58x4 | 232 | rps8/infA (IGR) |
| 16 | 98687-98716 | 15x2 | 30 | rps18 (CDS) |
| 17 | 112945-112986 | 21x2 | 42 | ycf1 (CDS) |
| 18 | 114040-114096 | 27x2 | 54 | ycf1 (CDS) |
| 19 | 121354-121425 | 21x3 | 63 | clpP/chlN (IGR) |
| 20 | 130208-130233 | 12x2 | 24 | trnK-UUU/ chlB (IGR) |
| 21 | 130205-130329 | 54x2 | 108 | trnK-UUU/ chlB (IGR) |
| 22 | 130262-130287 | 12x2 | 24 | trnK-UUU/ chlB (IGR) |
| 23 | 131093-131602 | 178x2 | 356 | trnK-UUU/ chlB (IGR) |
| 24 | 131255-131286 | 16x2 | 32 | trnK-UUU/ chlB (IGR) |
| 25 | 131433-131464 | 16x2 | 32 | trnK-UUU/ chlB (IGR) |

**Table S7** Characteristics of simple sequence repeats identified in the plastomes of *T. occidentalis*

|  | Mono | Di | Tri | Tetra | Penta | Hexa | Hepta | Octa | Nona | Total |
| --- | --- | --- | --- | --- | --- | --- | --- | --- | --- | --- |
| Total counts | 266 | 62 | 67 | 69 | 92 | 104 | 29 | 8 | 20 | 717 |
| Total Repeat Length (repeat unit X number of repeat) (bp) | 208 | 553 | 678 | 724 | 1085 | 1307 | 429 | 136 | 419 | 7416 |
| Density (Total repeat length/genome size) [bp/kb] | 15.84 | 4.20 | 5.15 | 5.50 | 8.24 | 9.93 | 3.26 | 1.03 | 3.18 | 56.33 |
| Proportion among other SSR (%) | 28.11 | 7.46 | 9.14 | 9.76 | 14.63 | 17.62 | 5.78 | 1.83 | 5.65 | 100 |
| Mean Length | 7.84 | 8.92 | 10.12 | 10.49 | 11.79 | 12.57 | 14.79 | 17.00 | 20.95 | 114.47 |

**Table S8** Distribution of tandem repeats in the *Thuja koraiensis*

| S/N | Indices | Size of repeat unit X Copy number | Repeat length | Location |
| --- | --- | --- | --- | --- |
| 1 | 4663-4755 | 45x2 | 90 | trnK-UUU/chlB (IGR) |
| 2 | 5715-5778 | 32x2 | 64 | trnK-UUU/chlB (IGR) |
| 3 | 5890-5953 | 32x2 | 64 | trnK-UUU/chlB (IGR) |
| 4 | 7910-7941 | 16x2 | 32 | trnQ-UUG/ trnT-UGU (IGR) |
| 5 | 18565-18595 | 16x2 | 32 | psbZ/ trnS-UGA (IGR) |
| 6 | 21701-21740 | 20x2 | 40 | psbD/ trnT-GGU (IGR) |
| 7 | 24254-24281 | 11x2 | 22 | trnD-GUC/ psbM (IGR) |
| 8 | 25392-25416 | 12x2 | 24 | petN/ trnC-GCA (IGR) |
| 9 | 45968-46013 | 24x2 | 48 | trnL-UAA/ trnF-GAA IGR) |
| 10 | 54987- 55012 | 12x2 | 24 | rpl32/ndhF (IGR) |
| 11 | 64281-64326 | 21x2 | 42 | trnI-GAU (IntronR) |
| 12 | 65201- 65246 | 23x2 | 46 | trnI-GAU/ rrn16 (IGR) |
| 13 | 69951-70039 | 21x4 | 84 | rps7/ ndhB (IGR) |
| 14 | 73262-73397 | 67x2 | 201 | trnI-CAU/ ycf2 (IGR) |
| 15 | 77227- 77269 | 21x2 | 42 | ycf2 (CDS) |
| 16 | 87699-87854 | 58x2 | 116 | rps8/ infA (IGR) |
| 17 | 99308-99339 | 16x2 | 32 | petA/ psbJ (IGR) |
| 18 | 105010-105055 | 23x2 | 46 | rps12/ ndhJ (IGR) |
| 19 | 111056-111095 | 20x2 | 40 | atpE/ rbcL (IGR) |
| 20 | 116267-116307 | 14x2 | 28 | chlN/ ycf1 (IGR) |
| 21 | 117344-117372 | 12x2 | 24 | ycf1 (CDS) |
| 22 | 118504-118560 | 27x2 | 54 | ycf1 (CDS) |
| 23 | 125877-125907 | 15x2 | 30 | clpP/ chlL (IGR) |
| 24 | 126105-126151 | 24x2 | 48 | clpP/ chlL (IGR) |

**Table S9** Characteristics of simple sequence repeats identified in the plastomes of *T. koraiensis*

|  | Mono | Di | Tri | Tetra | Penta | Hexa | Hepta | Octa | Nona | Total |
| --- | --- | --- | --- | --- | --- | --- | --- | --- | --- | --- |
| Species Total counts | 260 | 64 | 63 | 66 | 90 | 107 | 28 | 7 | 15 | 700 |
| Total Repeat Length (repeat unit X number of repeat) (bp) | 2074 | 589 | 659 | 701 | 1057 | 1346 | 399 | 125 | 303 | 7253 |
| Density (Total repeat length/genome size) [bp/kb] | 15.92 | 4.52 | 5.08 | 5.38 | 8.11 | 10.33 | 3.06 | 0.96 | 2.33 | 55.69 |
| Proportion among other SSR (%) | 28.60 | 8.12 | 9.09 | 9.66 | 14.57 | 18.56 | 5.50 | 1.72 | 4.18 | 100 |
| Mean Length | 7.98 | 9.20 | 10.46 | 10.62 | 11.74 | 12.57 | 14.25 | 17.85 | 20.20 | 114.87 |

**Table S10** Distribution of tandem repeats in the *Thuja standishii*

| S/N | Indices | Size of repeat unit X Copy number | Repeat length | Location |
| --- | --- | --- | --- | --- |
| 1 | 4545-4570 | 12x2 | 24 | trnK-UUU/chlB (IGR) |
| 2 | 5738-5787 | 18x2 | 36 | trnK-UUU/chlB (IGR) |
| 3 | 5888-5923 | 18x2 | 36 | trnK-UUU/chlB (IGR) |
| 4 | 7720-7751 | 16x2 | 32 | trnQ-UUG/trnT-UGU (IGR) |
| 5 | 18673-18699 | 13x2 | 26 | trnS-UGA/psbC (IGR) |
| 6 | 21505- 21544 | 20x2 | 40 | psbD/trnT-GGU (IGR) |
| 7 | 25529-25553 | 12x2 | 24 | petN/trnC-GCA (IGR) |
| 8 | 46090-46135 | 24x2 | 48 | trnL-UAA/trnF-GAA (IGR) |
| 9 | 55078-55103 | 12x2 | 24 | rpl32/ndhF (IGR) |
| 10 | 63942-63987 | 21x2 | 42 | trnI-GAU(IntronR) |
| 11 | 67129-67168 | 19x2 | 38 | rrn16/trnV-GAC (IGR) |
| 12 | 69589-69635 | 21x2 | 42 | rps7/ndhB (IGR) |
| 13 | 72858-72993 | 67x2 | 134 | trnI-CAU/ycf2 (IGR) |
| 14 | 74755-74786 | 15x2 | 30 | ycf2 (CDS) |
| 15 | 76787-76829 | 21x2 | 42 | ycf2 (CDS) |
| 16 | 83882-83929 | 24x2 | 48 | rpl22 (CDS) |
| 17 | 87325-87535 | 58x3 | 174 | rps8/infA (IGR) |
| 18 | 103295-103339 | 15x3 | 45 | rps18 (CDS) |
| 19 | 103428-103453 | 11x2 | 22 | rps18/rpl20 (IGR) |
| 20 | 107292-107349 | 28x2 | 56 | ndhC/trnV-UAC (IGR) |
| 21 | 113230-113289 | 30x2 | 60 | rbcL (IGR)/accD (CDS) |
| 22 | 117314-117357 | 12x3 | 36 | ycf1 (CDS) |
| 23 | 117399-117442 | 21x2 | 42 | ycf1 (CDS) |
| 24 | 118498-118581 | 27x3 | 81 | ycf1 (CDS) |
| 25 | 119023-119046 | 12x2 | 24 | ycf1 (CDS) |
| 26 | 126351-126397 | 24x2 | 48 | clpP/chlL (IGR) |

**Table: S11** Characteristics of simple sequence repeats identified in the plastomes of *T. standishii*

| Species | Mono | Di | Tri | Tetra | Penta | Hexa | Hepta | Octa | Nona | Total |
| --- | --- | --- | --- | --- | --- | --- | --- | --- | --- | --- |
| Total counts | 266 | 62 | 63 | 67 | 88 | 119 | 31 | 8 | 19 | 723 |
| Total Repeat Length (repeat unit X number of repeat) (bp) | 2128 | 570 | 656 | 711 | 1034 | 1493 | 460 | 67 | 419 | 7538 |
| Density (Total repeat length/genome size) [bp/kb] | 16.31 | 4.37 | 5.03 | 5.45 | 7.92 | 11.44 | 3.52 | 0.51 | 3.21 | 57.76 |
| Proportion among other SSR (%) | 28.23 | 7.56 | 8.70 | 9.43 | 13.72 | 19.81 | 6.10 | 0.89 | 5.56 | 100 |
| Mean Length | 8.00 | 9.19 | 10.41 | 10.61 | 11.75 | 12.54 | 14.84 | 8.38 | 22.05 | 107.77 |

**Table: S12** The dispersal matrices for the different time slices appied in the ancestral area reconstruction. Four time periods was defined, 0-1: 0-5 Ma, 1-2: 5-30 Ma, 2-3: 30-45 Ma, and 3-4: 45-65 Ma

| **Time periods** | **A** | **B** | **C** | **D** |
| --- | --- | --- | --- | --- |
| **0-1 A** | 1 | 0.5 | 0.1 | 0.1 |
| **0-1 B** | 0.5 | 1 | 0.25 | 0.25 |
| **0-1 C** | 0.1 | 0.25 | 1 | 1 |
| **0-1 D** | 0.1 | 0.25 | 1 | 1 |
| **1-2 A** | 1 | 0.5 | 1 | 1 |
| **1-2 B** | 0.5 | 1 | 0.75 | 0.75 |
| **1-2 C** | 1 | 0.75 | 1 | 1 |
| **1-2 D** | 1 | 0.75 | 1 | 1 |
| **2-3 A** | 1 | 0.5 | 0.1 | 0.1 |
| **2-3 B** | 0.5 | 1 | 0.25 | 0.25 |
| **2-3 C** | 0.1 | 0.25 | 1 | 1 |
| **2-3 D** | 0.1 | 0.25 | 1 | 1 |
| **3-4 A** | 1 | 0.5 | 1 | 1 |
| **3-4 B** | 0.5 | 1 | 0.75 | 0.75 |
| **3-4 C** | 1 | 0.75 | 1 | 1 |
| **3-4 D** | 1 | 0.75 | 1 | 1 |
